# Supplementary material for: Integrated Omic Approaches Reveal Molecular Mechanisms of Tolerance during Soybean and Meloidogyne incognita Interactions
Source: Plants (Basel). 2022 Oct 17;11(20):2744. doi: 10.3390/plants11202744 (PMC9612212; doi:10.3390/plants11202744)
Supplement: Supplementary file 1 [file Figure S6.pdf › plants-1905218-supplementary/Figure S6.pdf]

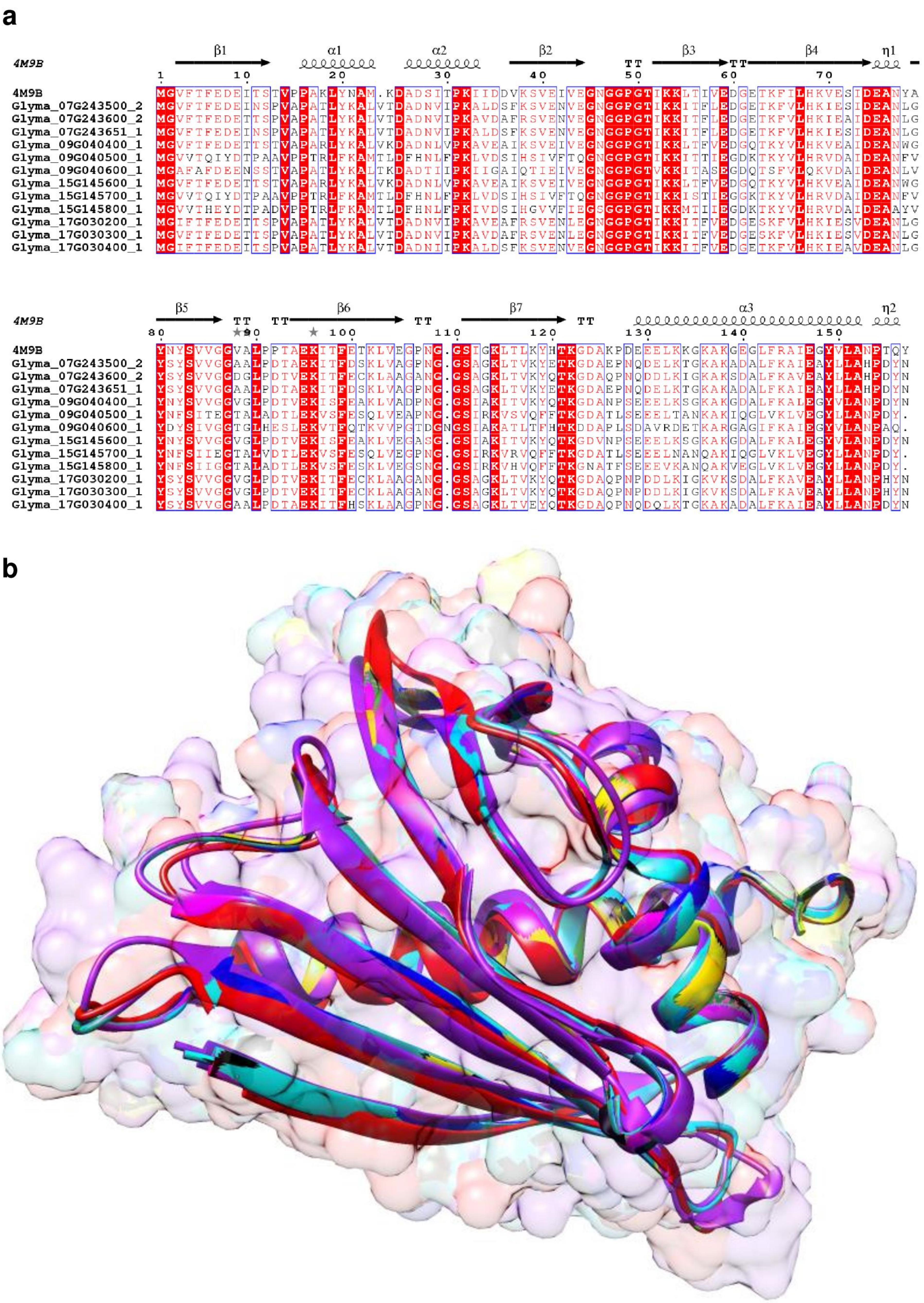

Figure S6. *In silico* analysis of soybean GmPR10 protein and its paralogs. (**a**) Protein alignment with all 11 paralogs of GmPR10 (Glyma.17G030400.1) selected in the Wm82.a2.v1 reference genome assembly of *Glycine max* in Phytozome v.13 database. The graphical representation of the alignment was made with ESPript version 3 online software (https://espript.ibcp.fr/) using as a model for the secondary structure representation (top on the alignment) the structure of the panallergen Ara h 8 from peanuts (*Arachis hypogaea*; PDB ID: 4M9B), used to generate structural model, as follows. (**b**) Superposition of the structural models of the GmPR10 protein and its 11 paralogs, obtained by similarity. Parameters analyzed to choose the best model: *GMQE* (*Global Model Quality Estimate*) – 0.81-0.86; *Coverage* – greater than 0.93; *QMEAN DisCO Global* (*Qualitative Model Energy Analysis*) – 0.86 ± 0.07. *Color Legend* (structural models): Glyma.07G243500.2 – *hot pink*; Glyma.07G243600.2 – *yellow*; Glyma.07G243651.1 – *green*; Glyma.09G040400.1 – *cyan*; Glyma.09G040500.1 – *blue*; Glyma.09G040600.1 – *medium blue*; Glyma.15G145600.1 – purple; Glyma.15G145700.1 – *red*; Glyma.15G145800.1 – *light green*; Glyma.17G030200.1 – *dark gray*; Glyma.17G030300.1 – *magenta*; Glyma.17G030400.1 – *black*.
