# Supplementary material for: Integrated Omic Approaches Reveal Molecular Mechanisms of Tolerance during Soybean and Meloidogyne incognita Interactions
Source: Plants (Basel). 2022 Oct 17;11(20):2744. doi: 10.3390/plants11202744 (PMC9612212; doi:10.3390/plants11202744)
Supplement: Supplementary file 1 [file plants-11-02744-s001.zip › plants-1905218-supplementary/Figure S1.pdf]

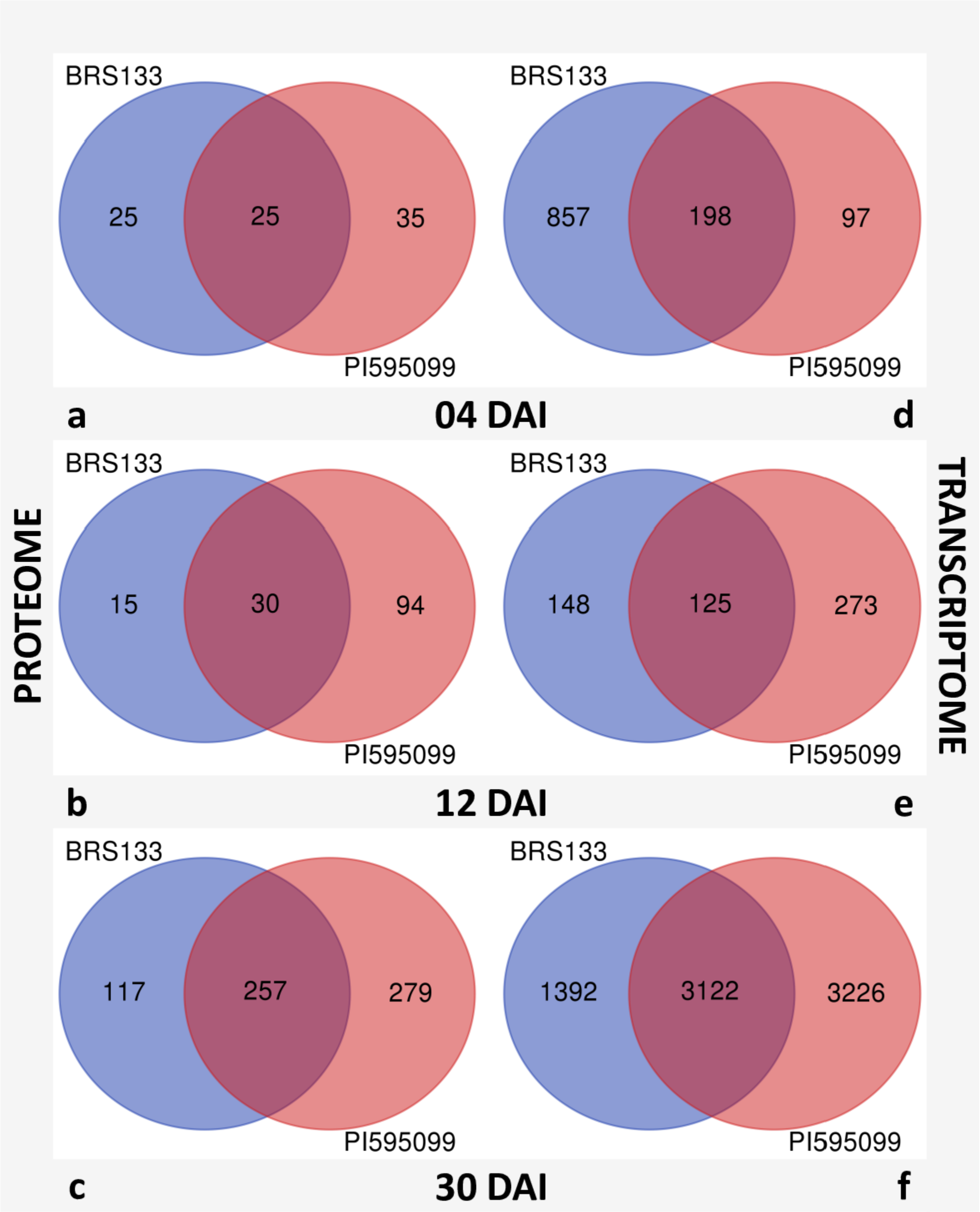

Figure S1. Differently expressed genes (DEGs) and proteins (DEPs) between soybean genotypes. The Venn diagrams show the distribution of DEPs (**a** - **c**) and DEGs (**d** - **f**) on different days after inoculation (DAI) with *Meloidogyne incognita*: 4 DAI (**a**, **d**), 12 DAI (**b**, **e**) and 30 DAI (**c**, **f**). It is possible to observe the number of proteins or transcripts that are expressed exclusively in each soybean genotype (BRS 133 and PI 595099), as well as those that are shared (interceptions).
