# Supplementary material for: Integrated Omic Approaches Reveal Molecular Mechanisms of Tolerance during Soybean and Meloidogyne incognita Interactions
Source: Plants (Basel). 2022 Oct 17;11(20):2744. doi: 10.3390/plants11202744 (PMC9612212; doi:10.3390/plants11202744)
Supplement: Supplementary file 1 [file plants-11-02744-s001.zip › plants-1905218-supplementary/Figure S2.pdf]

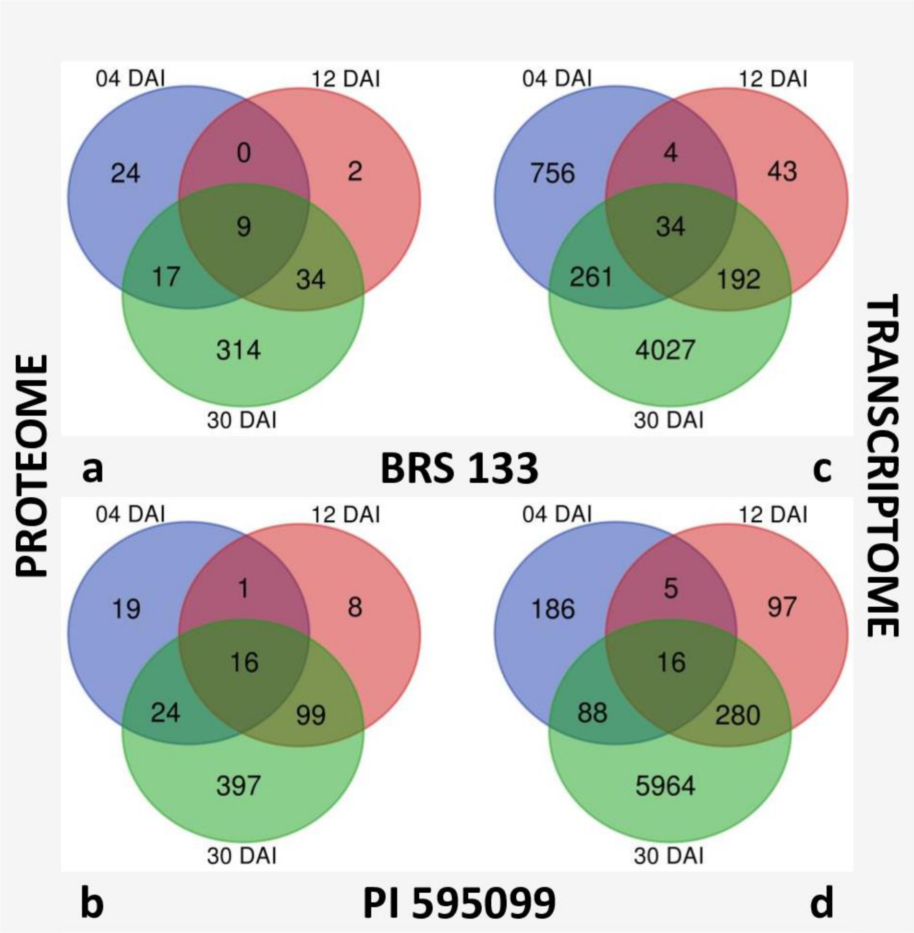

Figure S2. Differentially expressed genes (DEGs) and proteins (DEPs) in soybean genotypes over time. The Venn diagrams show the distribution of DEPs (**a, b**) and DEGs (**c**, **d**) in soybean genotypes: BRS 133 (**a**, **c**) and PI 595099 (**b**, **d**), during the inoculation with the nematode *Meloidogyne incognita*. It is possible to observe the number of proteins or transcripts that are expressed exclusively each day after infection (4, 12 and 30 DAI: days after inoculation), as well as those that are shared (interceptions).
