# Supplementary material for: Integrated Omic Approaches Reveal Molecular Mechanisms of Tolerance during Soybean and Meloidogyne incognita Interactions
Source: Plants (Basel). 2022 Oct 17;11(20):2744. doi: 10.3390/plants11202744 (PMC9612212; doi:10.3390/plants11202744)
Supplement: Supplementary file 1 [file plants-11-02744-s001.zip › plants-1905218-supplementary/Figure S3.pdf]

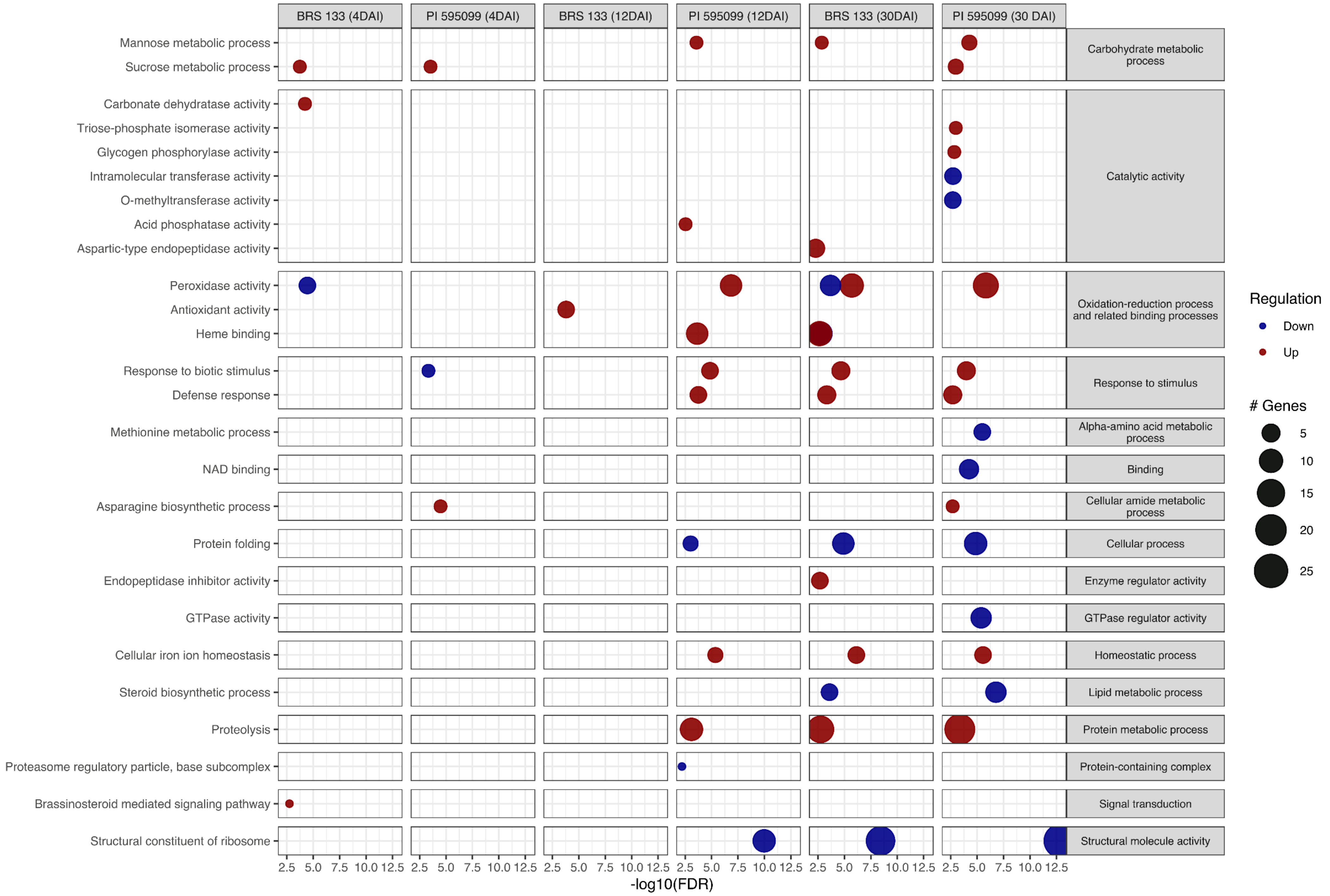

Figure S3. Enrichment of Gene Ontology (GO) terms in the proteome of *Glycine max* challenged with *Meloidogyne incognita*. The susceptible (BRS 133) and the tolerant (PI 595099) genotypes are represented at the top of the bubble chart, followed by the number of days after inoculation (DAI) with *M. incognita*. The GO terms are listed on the left (y-axis). Only GO terms with statistical significance (false discovery rate, FDR > 0.05) were included.
