# Supplementary material for: Integrated Omic Approaches Reveal Molecular Mechanisms of Tolerance during Soybean and Meloidogyne incognita Interactions
Source: Plants (Basel). 2022 Oct 17;11(20):2744. doi: 10.3390/plants11202744 (PMC9612212; doi:10.3390/plants11202744)
Supplement: Supplementary file 1 [file plants-11-02744-s001.zip › plants-1905218-supplementary/Figure S4.pdf]

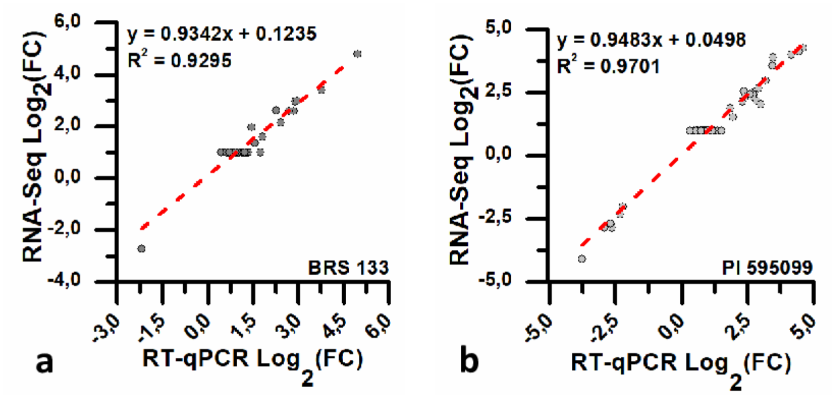

Figure S4. Correlation between RNA-Seq and RT-qPCR analyses. The graphs represent the $\log_2$ (FC; fold-change) between the RNA-Seq and RT-qPCR data of the 20 genes validated in soybean genotypes BRS 133 (**a**) and PI 595099 (**b**). The red dotted line represents the trend line of the plotted data, which was used to determine the equation of the line and R^2. Thus, the correlation of the data in cultivar BRS 133 was 92.95% and for PI 595099 was 97.01%.
