# Supplementary material for: Integrated Omic Approaches Reveal Molecular Mechanisms of Tolerance during Soybean and Meloidogyne incognita Interactions
Source: Plants (Basel). 2022 Oct 17;11(20):2744. doi: 10.3390/plants11202744 (PMC9612212; doi:10.3390/plants11202744)
Supplement: Supplementary file 1 [file plants-11-02744-s001.zip › plants-1905218-supplementary/Figure S5.pdf]

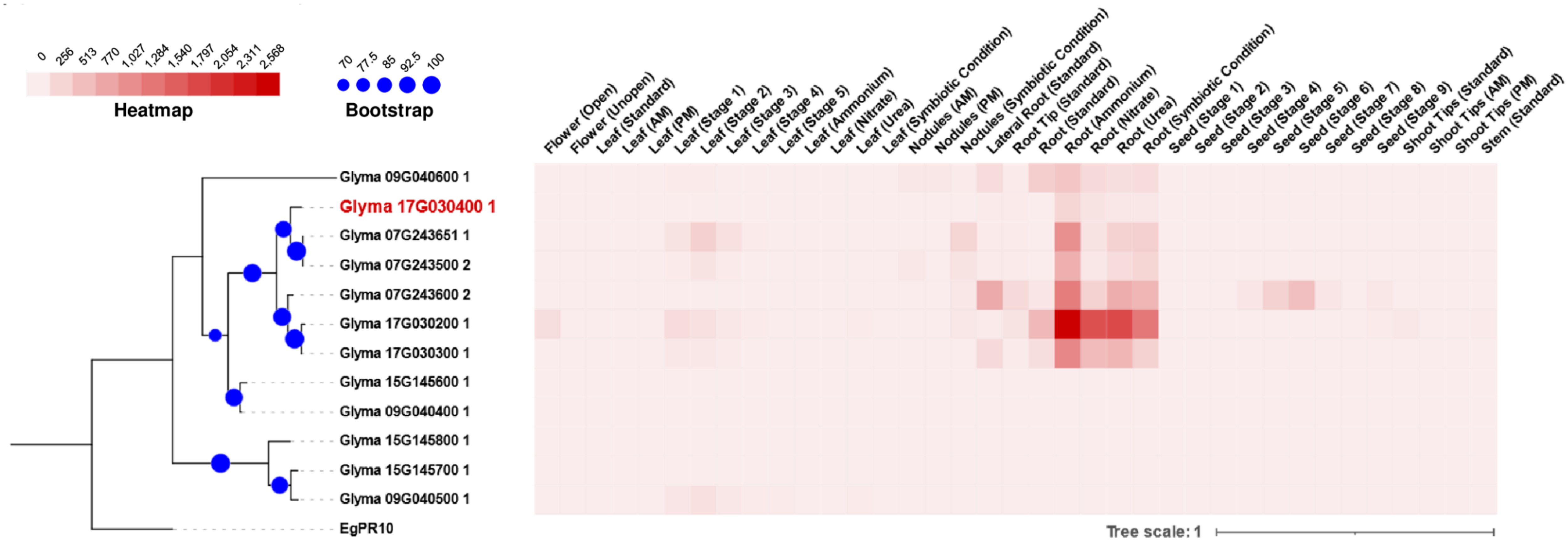

Figure S5. Phylogenetic relationships and expression profiles of soybean *GmPR10* and its paralogs. Maximum likelihood analysis shows the clustering of the GmPR10 protein (Glyma.17G030400.1) with its 11 paralogs, all sharing the same clade, which demonstrates the intrinsic and close phylogenetic relationship between the proteins evaluated. The selected outgroup was the putative GmPR10 ortholog encoded by *Eucalyptus grandis* (EgPR10; NCBI XP_010064159_2). Bootstrap values are presented in scale (blue circles), ranging from 70 (smaller circles) to 100 (larger circles). Bootstrap values lower than 70 were omitted from the phylogenetic tree. On the right, the heatmap shows the transcript expression profile [$\log_2$ (FPKM)] of *GmPR10* and its paralogs analyzed from soybean Gene Atlas version 2 available in the Phytozome version 13 database and viewed with the Phytomine online platform (http://phytozome.jgi.doe.gov/phytomine/). The tissue samples and conditions used for expression profile were root: root, lateral root, root tip, root hairs, nodules, shoot tip, apical meristem shoot, leaf, flower, and seed in a series of developmental stages, as well as opened and unopened flower, root and leaf varied by nitrogen source and symbiotic conditions for root, nodules, and leaf. The color scale shown above represents the expression levels, where light pink indicates the lowest expression level (zero) and red indicates the highest transcript abundance levels (2,568).
