# Supplementary material for: Integrated Omic Approaches Reveal Molecular Mechanisms of Tolerance during Soybean and Meloidogyne incognita Interactions
Source: Plants (Basel). 2022 Oct 17;11(20):2744. doi: 10.3390/plants11202744 (PMC9612212; doi:10.3390/plants11202744)
Supplement: Supplementary file 1 [file plants-11-02744-s001.zip › plants-1905218-supplementary/Figure S7.pdf]

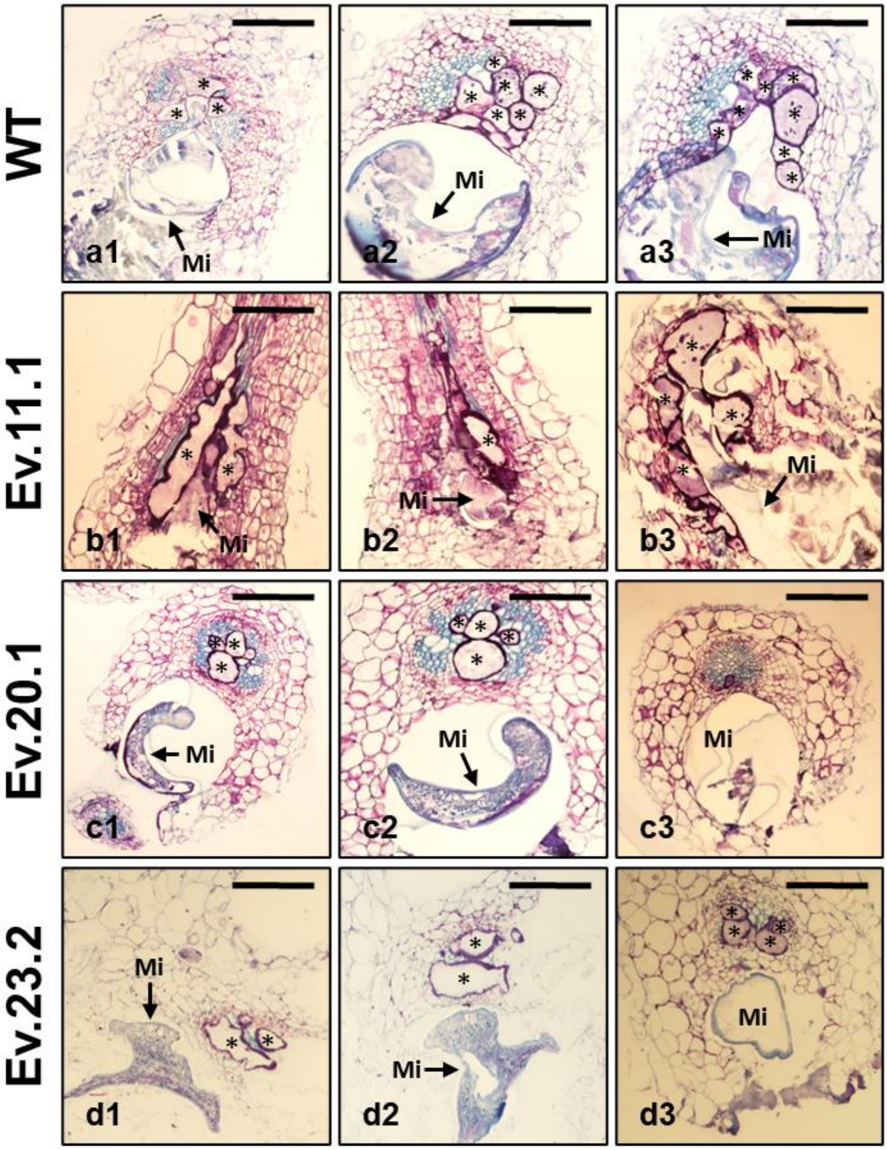

Figure S7. Complementary histological analysis of *M. incognita*-induced galls in transgenic *N. tabacum* overexpressing the soybean *GmPR10* gene. Similar to the data presented in Fig. 8, at least five biological replicates from each line were infected with *M. incognita*, and gall and nematode morphology were evaluated 60 days after inoculation (DAI). (**a1-a3**) non-transformed wild-type (WT); (**b1-b3**) E11.1, (**c1-c3**) E20.1 and E23.2 (**d1-d3**). The three slides presented for each condition represent different sections of the same sample. Legend: (*) *giant cell*; (Mi) *M. incognita*. 50 µm.
