# Supplementary material for: Integrated Omic Approaches Reveal Molecular Mechanisms of Tolerance during Soybean and Meloidogyne incognita Interactions
Source: Plants (Basel). 2022 Oct 17;11(20):2744. doi: 10.3390/plants11202744 (PMC9612212; doi:10.3390/plants11202744)
Supplement: Supplementary file 1 [file plants-11-02744-s001.zip › plants-1905218-supplementary/Table S2.pdf]

Table S2. Differentially expressed genes with opposite expression between tolerant (PI 595099) and susceptible (BRS 133) genotypes in at least one condition after inoculation with *M. incognita*

**Overexpressed in PI 595099 and Underexpressed in BRS 133**	
**Genome ID**	**Predicted protein (PFAM)**
Glyma.01G025700	NA - Not annotated (without match in the protein database)
Glyma.01G027200	NA - Not annotated (without match in the protein database)
Glyma.02G135100	Peroxidase
Glyma.02G247100	Myb-like DNA-binding domain
Glyma.02G300500	NUDIX domain
Glyma.03G061700	Starch binding domain
Glyma.03G096500	2OG-Fe(II) oxygenase superfamily
Glyma.03G171600	Sodium/hydrogen exchanger family
Glyma.03G171700	Sodium/hydrogen exchanger family
Glyma.03G206800	Alpha/beta hydrolase fold
Glyma.03G224300	SET domain
Glyma.03G246800	EF hand
Glyma.04G024700	NA - Not annotated (without match in the protein database)
Glyma.04G035600	Cytochrome P450
Glyma.04G197700	S locus-related glycoprotein 1 binding pollen coat protein (SLR1-BP)
Glyma.05G005800	Glycosyl hydrolases family 28
Glyma.06G043000	Asparaginase
Glyma.06G099500	NA - Not annotated (without match in the protein database)
Glyma.06G115800	Natural resistance-associated protein
Glyma.06G307900	Cellulose synthase
Glyma.07G048800	O-methyltransferase; Dimerization domain
Glyma.07G067700	Ferric reductase like transmembrane protein
Glyma.07G124400	2OG-Fe(II) oxygenase superfamily
Glyma.07G209900	Peroxidase
Glyma.08G006300	Amidase
Glyma.08G065300	K-box region; SRF-type transcription factor
Glyma.08G169100	2OG-Fe(II) oxygenase superfamily
Glyma.08G175400	Nicotianamine synthase protein
Glyma.08G338800	UDP-glucoronosyl and UDP-glucosyl transferase
Glyma.08G356900	Helix-loop-helix DNA-binding domain
Glyma.10G004200	Protein phosphatase 2C
Glyma.10G021000	Glutaredoxin
Glyma.12G069600	NA - Not annotated (without match in the protein database)
Glyma.13G252200	NA - Not annotated (without match in the protein database)
Glyma.13G299100	Protein of unknown function (DUF789)
Glyma.14G206600	Cation efflux family
Glyma.15G223900	NADH: flavin oxidoreductase
Glyma.15G251300	Nicotianamine synthase protein
Glyma.16G177800	Protein of unknown function (DUF3464)
Glyma.17G046600	Flavin-binding monooxygenase-like
Glyma.18G037600	PAN-like domain; D-mannose binding lectin
Glyma.19G161400	Photosystem II reaction center W protein (PsbW)
Glyma.20G194600	Zinc finger, C3HC4 type (RING finger)

Table S2 (cont.). Differentially expressed genes with opposite expression between tolerant (PI 595099) and susceptible (BRS 133) genotypes in at least one condition after inoculation with *M. incognita*

**Overexpressed in BRS 133 and Underexpressed in PI 595099**	
**Genome ID**	**Predicted protein (PFAM)**
Glyma.02G004800	NA - Not annotated (without match in the protein database)
Glyma.02G154200	K^+ potassium transporter
Glyma.02G252800	Hsp20/alpha crystallin family
Glyma.03G115200	Plant protein of unknown function
Glyma.03G222400	NA - Not annotated (without match in the protein database)
Glyma.04G028700	NA - Not annotated (without match in the protein database)
Glyma.04G108500	Hsp20/alpha crystallin family
Glyma.05G041100	Universal stress protein family
Glyma.06G047300	NA - Not annotated (without match in the protein database)
Glyma.06G101100	NA - Not annotated (without match in the protein database)
Glyma.06G119200	Calcium-binding EGF domain
Glyma.07G038700	Cupin
Glyma.07G043600	Hsp20/alpha crystallin family
Glyma.08G160800	NA - Not annotated (without match in the protein database)
Glyma.08G212000	Hsp20/alpha crystallin family
Glyma.08G268400	Protease inhibitor/seed storage/LTP family
Glyma.08G359200	Multicopper oxidase;
Glyma.09G023500	Plant self-incompatibility protein S1
Glyma.09G059500	ANTH domain
Glyma.09G168300	Glycosyl hydrolase family 14
Glyma.10G017100	Pectinesterase; Plant invertase/pectin methylesterase inhibitor
Glyma.10G176700	Dimerization domain; O-methyltransferase
Glyma.10G283500	Protein phosphatase 2A regulatory B subunit (B56 family)
Glyma.12G046200	NmrA-like family
Glyma.12G053300	NA - Not annotated (without match in the protein database)
Glyma.12G075600	Senescence regulator
Glyma.13G062500	Dof domain, zinc finger
Glyma.13G164700	NA - Not annotated (without match in the protein database)
Glyma.13G203000	MatE
Glyma.13G302400	Myb-like DNA-binding domain
Glyma.13G363300	NA - Not annotated (without match in the protein database)
Glyma.14G001600	RWP-RK domain; PB1 domain
Glyma.14G091000	Homeobox associated leucine zipper; Homeobox domain
Glyma.14G162100	Protein phosphatase 2C
Glyma.15G026300	Lipoxygenase; PLAT/LH2 domain
Glyma.15G111100	NA - Not annotated (without match in the protein database)
Glyma.15G242700	Triose-phosphate Transporter family
Glyma.16G017500	2OG-Fe(II) oxygenase superfamily
Glyma.16G065600	Leucine Rich Repeat; Leucine rich repeat N-terminal domain
Glyma.16G100100	Protease inhibitor/seed storage/LTP family
Glyma.17G040800	Late embryogenesis abundant protein
Glyma.17G080500	Cytochrome P450
Glyma.18G055300	Peroxidase
Glyma.18G063200	Sodium/calcium exchanger protein
Glyma.18G236200	Nop14-like family
Glyma.18G273400	Myb-like DNA-binding domain
Glyma.19G012200	NA - Not annotated (without match in the protein database)
Glyma.19G069200	Protein phosphatase 2C
Glyma.19G189400	Late embryogenesis abundant protein
Glyma.19G217700	Raffinose synthase or seed imbibition protein Sip1
Glyma.20G011200	GRAM domain
Glyma.20G183900	NA - Not annotated (without match in the protein database)
