# Supplementary material for: Integrated Omic Approaches Reveal Molecular Mechanisms of Tolerance during Soybean and Meloidogyne incognita Interactions
Source: Plants (Basel). 2022 Oct 17;11(20):2744. doi: 10.3390/plants11202744 (PMC9612212; doi:10.3390/plants11202744)
Supplement: Supplementary file 1 [file plants-11-02744-s001.zip › plants-1905218-supplementary/Table S3.pdf]

Table S3. Predicted genes commonly found as differentially expressed by transcriptomics (Transcript) and proteomics (Protein) analyses. Statistically significant values of log_2(FC; fold-change) are given for each gene in its respective treatment (DAI: days after inoculation with *M. incognita*)

Underexpressed Genes													
Genome ID	Description	30 DAI				12 DAI				4 DAI			
		Transcript		Protein		Transcript		Protein		Transcript		Protein	
		PI 595099	BRS 133	PI 595099	BRS 133	PI 595099	BRS 133	PI 595099	BRS 133	PI 595099	BRS 133	PI 595099	BRS 133
Glyma.01G046300	Glucosyl/glucuronosyl transferase	-4.5	-7.5	-1.7	-1.0								
Glyma.01G083300	Single-stranded endonuclease	-2.4	-6.8	-1.2	-2.3								
Glyma.01G126600	Dirigent protein 19	-3.4	-7.1	-3.4	-1.2								
Glyma.01G167500	GDP-mannose 4,6-dehydratase	-2.1		-2.0	-2.1								
Glyma.01G172700	NAD dependent epimerase/dehydratase		-6.5		-1.2								
Glyma.01G215500	Hydroxymethylglutaryl-CoA synthase	-3.0		-1.6	-2.2			-1.2					
Glyma.02G104600	Glucosyl/glucuronosyl transferase	-2.7		-3.0	-2.1								
Glyma.02G115900	14-3-3-like protein GF14 (IOTA)	-2.2		-1.7	-1.3								
Glyma.02G151700	Peroxidase/Lactoperoxidase	-6.6	-9.9		-1.0								-1.0
Glyma.02G270200	Niemann pick type C2 protein (NPC2)	-2.2		-2.1									
Glyma.02G277300	Late embryogenesis abundant protein (LEA2)	-2.0		-2.7									
Glyma.03G029400	Pectinesterase inhibitor 17-related	-5.7	-9.6		-1.0								
Glyma.03G030500	Chitinase	-3.7	-6.8	-1.0	-1.6								
Glyma.03G124400	Tubulin beta-6 chain	-3.2	-6.7	-2.2	-1.9								
Glyma.03G208200	Peroxidase/Lactoperoxidase	-5.8	-8.2		-2.3					-2.5	-2.7		-1.0
Glyma.03G232000	Protein disulfide isomerase	-3.5	-6.3		-1.5								
Glyma.03G259600	CYPB5A	-2.3		-1.4									
Glyma.04G040000	Transferase family		-6.6		-1.8								
Glyma.04G107900	Universal stress protein family (USP)	-2.7		-1.3									
Glyma.04G227700	Flavone 3'-O-methyltransferase 1	-2.5		-1.4									
Glyma.05G056400	Transaldolase-like protein	-3.0	-6.7	-1.2									
Glyma.05G103600	Peroxidase 35	-3.6	-6.6	-1.0									
Glyma.05G110200	Tubulin alpha-2 chain	-2.0		-2.0									
Glyma.05G152100	Serine hydroxy methyltransferase	-3.1	-6.5	-1.9									
Glyma.05G207500	Tubulin	-2.5	-1.9	-2.5	-1.3								
Glyma.05G231800	Aldehyde dehydrogenase family	-4.7	-9.1		-1.1								
Glyma.05G247300	Inactive receptor kinase (RLK902)	-2.4		-1.6	-1.3								
Glyma.06G056000	NA - Not annotated (without match in the protein database)	-4.1	-7.1	-2.0	-1.1								
Glyma.06G087800	NADP dependent malic enzyme 2-related		-7.7	-3.1	-1.9			-1.1	-1.1				
Glyma.06G205600	UDP-arabinopyranose mutase 3	-2.1		-1.7									
Glyma.06G229900	Phosphoenolpyruvate carboxylase	-2.9	-6.9	-1.6	-1.2								
Glyma.06G302600	Peroxidase 3	-4.6		-1.2								-1.1	-1.3
Glyma.07G033700	2OG-Fe(II) oxygenase superfamily	-3.3	-6.6	-2.7	-2.6					-2.0		-1.4	-1.4
Glyma.07G038600	Cupin domain	-3.5	-6.5	-1.7	-1.0								
Glyma.07G087500	Aldehyde dehydrogenase family	-2.6	-6.4	-1.4	-1.5								
Glyma.07G140400	Glutathione S-transferase (GST)		-6.3		-1.1								
Glyma.07G162900	CYP450	-2.4	-6.5		-1.4								
Glyma.07G241600	Lipoyl dehydrogenase	-3.0	-7.0	-1.9									
Glyma.07G254600	UDP-glucosyl transferase (73B2)	-2.9		-1.5	-1.3								
Glyma.07G256100	Lysozyme/Muramidase	-3.2	-7.5	-1.8	-2.9								
Glyma.08G137000	Expressed protein-related	-4.6	-7.2										-1.1
Glyma.08G181000	Glucosyl/glucuronosyl transferase	-2.2		-1.5									
Glyma.08G189600	Linoleate 9S-lipoxygenase	-4.7	-9.0	-2.4	-3.3								
Glyma.08G230100	Pathogenesis-related protein Bet v I family	-4.0	-7.1	-2.8	-2.0							-1.0	
Glyma.08G238100	CYP72A154	-2.5		-2.9	-2.1								
Glyma.09G007900	Diphosphate-fructose-6-phosphate 1-phosphotransferase	-3.7	-6.8	-2.1	-1.1								
Glyma.09G062900	Beta-galactosidase 1	-3.9	-7.6		-1.6							-1.3	-1.2

Table S3 (cont.). Predicted genes commonly found as differentially expressed by transcriptomics (Transcript) and proteomics (Protein) analyses. Statistically significant values of log_2(FC; Fold-change) are given for each gene in its respective treatment (DAI: days after inoculation with *M. incognita*)

Underexpressed Genes (cont.)													
Genome ID	Description	30 DAI				12 DAI				4 DAI			
		Transcript		Protein		Transcript		Protein		Transcript		Protein	
		PI 595099	BRS 133	PI 595099	BRS 133	PI 595099	BRS 133	PI 595099	BRS 133	PI 595099	BRS 133	PI 595099	BRS 133
Glyma.09G149400	Carboxylesterase 9-related	-2.0	-7.1	-1.3									
Glyma.09G155500	DR4 protein-related	-2.9		-1.4	-1.1			-1.1					
Glyma.09G176100	Aldose-1-epimerase	-2.2	-6.8		-1.9								
Glyma.09G243700	Proprotein convertase subtilisin	-4.4	-7.5		-1.0								
Glyma.10G022500	Peroxidase/Lactoperoxidase	-5.9	-12.4		-1.4								
Glyma.10G058000	Adenosine kinase 1-related	-2.1		-1.1									
Glyma.10G104700	Glucosyl/glucuronosyl transferase	-3.1		-2.3	-1.6								
Glyma.10G147600	Calreticulin and Calnexin	-2.0			-1.9			-3.6					
Glyma.10G159600	Acetyltransferase	-2.0	-6.3		-1.6								
Glyma.10G167800	Ammonium transporter 1 member 2										-2.9		-1.6
Glyma.10G291100	Glutathione S-transferase (DHAR3)	-2.3		-1.5									
Glyma.11G053400	Glucosyl/glucuronosyl transferase	-2.7		-3.0	-1.3								
Glyma.11G103500	Lanosterol delta(24)-reductase	-3.0	-6.6	-3.1	-2.2								
Glyma.11G113000	Coatomer	-2.2		-2.3	-1.5								
Glyma.11G228000	Aquaporin (PIP1-5)	-2.6	-7.1		-1.2								
Glyma.11G247600	Glyceraldehyde-3-phosphate dehydrogenase (GAPC1)	-3.3	-6.9	-2.3	-1.9								
Glyma.12G019800	Alcohol dehydrogenase-related	-2.9	-7.4	-1.0	-2.2								
Glyma.12G101200	Peroxidase 3	-3.9		-1.5								-1.2	-1.1
Glyma.12G207600	Fasciclin-like arabinogalactan protein 13-related	-5.3	-7.8		-1.1								
Glyma.13G199800	Annexin	-3.0		-1.4									
Glyma.13G266100	Protein tyrosine kinase (Tyr)										-2.0		-1.2
Glyma.14G004600	Acetoacetyl-CoA thiolase	-2.1		-1.7	-1.2								
Glyma.14G037300	Late embryogenesis abundant protein (LEA2)	-2.9		-1.0									
Glyma.15G008800	Embryo-specific protein 3 (ATS3)	-2.1		-1.4								-2.7	
Glyma.15G121400	Farnesyl-pyrophosphate synthetase	-2.1		-1.2									
Glyma.15G238400	Annexin D4	-4.1	-6.8	-1.0	-1.6								
Glyma.15G251600	Glutathione S-transferase (U21)	-2.1	-6.3	-2.2	-1.5								
Glyma.16G033700	Glucosyl/glucuronosyl transferase	-2.3		-1.6	-1.1								
Glyma.16G164400	Peroxidase/Lactoperoxidase	-4.4	-7.6	-2.1	-2.6								
Glyma.16G211700	DR4 protein-related	-3.0		-2.5	-1.6								
Glyma.16G211800	DR4 protein-related	-4.3	-7.6	-2.7	-2.3								
Glyma.17G019300	Amine oxidase-related	-4.4	-7.5		-1.2								
Glyma.17G061400	Peroxidase 16	-4.2	-7.5		-1.2								
Glyma.17G132500	dTDP-4-dehydrorhamnose reductase	-2.7	-6.6	-3.1	-2.1								
Glyma.17G171300	Anthranilate N-hydroxycinnamoyl		-6.5	-1.8	-1.8								
Glyma.18G018600	Inositol-3-phosphate synthase	-2.1		-1.3									
Glyma.18G019600	Cellulose synthase-interactive protein 1	-2.4		-3.6	-1.4								
Glyma.18G091500	Alcohol dehydrogenase-related	-2.3		-1.7	-1.1								
Glyma.18G229800	Phosphomannomutase	-2.1		-2.2									
Glyma.18G258000	Isoflavone-7-O-beta-glucoside 6"-O-malonyltransferase	-2.2		-1.9	-1.4								
Glyma.18G285800	Aldo/keto reductase		-6.3		-1.3								
Glyma.19G008600	Enone oxidoreductase		-6.5		-1.5								
Glyma.19G028500	UDP-glucose dehydrogenase	-3.5		-3.5	-1.5								
Glyma.19G124600	Alpha-l-arabinofuranosidase 1-related	-3.1	-7.4										-1.1
Glyma.19G196900	KR domain (KR)	-2.9	-7.1	-1.5	-1.8							-1.2	-1.1
Glyma.20G015900	Hsp20 family	-2.4		-1.9								-1.9	
Glyma.20G159200	Tubulin	-2.1		-1.3									
Glyma.20G213700	Methyltransferase	-2.2	-6.5	-2.7	-2.6								

Table S3 (cont.). Predicted genes commonly found as differentially expressed by transcriptomics (Transcript) and proteomics (Protein) analyses. Statistically significant values of $\log_2$(FC; Fold-change) are given for each gene in its respective treatment (DAI: days after inoculation with *M. incognita*)

Overexpressed Genes													
Genome ID	Description	30 DAI				12 DAI				4 DAI			
		Transcript		Protein		Transcript		Protein		Transcript		Protein	
		PI 595099	BRS 133	PI 595099	BRS 133	PI 595099	BRS 133	PI 595099	BRS 133	PI 595099	BRS 133	PI 595099	BRS 133
Glyma.01G018000	NA - Not annotated (without match in the protein database)	2.4	2.6	3.1	4.3			1.9	1.3				
Glyma.01G124500	Ferritin	3.9	4.8	7.5	6.6	2.6		2.1			3.0		
Glyma.03G137900	Raffinose synthase										3.0	1.4	2.9
Glyma.03G216300	Sucrose synthase 2			1.8	1.8						2.3	1.3	1.2
Glyma.04G040000	Transferase family											1.7	
Glyma.04G240800	Alcohol dehydrogenase		2.2	1.3	1.6								
Glyma.05G003900	Galactinol-sucrose galactosyltransferase 5							1.2			4.3	3.0	1.5
Glyma.06G260200	Aldo/keto reductase	2.1		2.4	1.5			1.2					
Glyma.06G301500	Beta-amylase 5-related		3.0	2.4	6.5			2.2	3.0				
Glyma.07G155200	Ferritin			4.4	4.3			2.0	1.1		2.3		1.3
Glyma.07G243500	Pathogenesis-related protein Bet v I family	3.2		1.0							2.9		
Glyma.08G128100	Basic blue protein	2.7		4.1	3.2			1.7					
Glyma.08G200100	Acid phosphatase-related	4.0	3.6	4.6	3.9			3.2	2.9		2.1	1.3	
Glyma.08G208700	Molybdate transporter 2	2.1		4.3	1.7			1.2					
Glyma.09G139200	Remorin, N-terminal region (Remorin-N)	2.4		4.9	1.9			2.4					
Glyma.11G164700	Cinnamoyl-CoA reductase-like protein		2.2	2.3	3.3			1.5	1.4				
Glyma.13G088700	Annexin D1				1.2						3.4	1.5	1.0
Glyma.13G237000	Dormancy/auxin associated protein (auxin repressed)	2.4		2.1	2.0								
Glyma.14G195000	Asparagine synthase (glutamine-hydrolyzing)		2.3	2.0	1.9							1.2	
Glyma.15G071200	Catechol oxidase/Tyrosinase	2.0		2.8	2.2				1.3				
Glyma.15G211500	Trypsin and protease inhibitor (Kunitz legume)		2.7	3.6	3.3		2.0		1.6		3.6		2.0
Glyma.16G218500	Glutamate decarboxylase 2	2.4	2.7	4.3	4.0			2.6	2.2			3.0	
Glyma.17G030100	Pathogenesis-related protein Bet v I family		2.0	2.3	1.8			1.3					
Glyma.17G030400	Pathogenesis-related protein Bet v I family	2.9		4.1							2.6	1.9	
Glyma.18G027100	Phosphoinositide phospholipase C				1.1						3.3		
Glyma.18G190300	Glutathione S-transferase (GST)		2.0	1.9	2.2			1.0	1.0				
Glyma.19G006200	Pectin lyase-like superfamily protein		2.1	1.3	2.0	2.1		1.7	1.2				
Glyma.19G008500	Enone oxidoreductase		3.3	3.5	6.8			3.3	4.5			3.3	3.2
Glyma.19G009000	2-hydroxyacid dehydrogenase-related			3.9	2.6			1.3			2.7		
Glyma.19G114700	NA - Not annotated (without match in the protein database)				2.7						3.0		2.8
Glyma.19G211000	Adenine nucleotide alpha hydrolases-like protein		2.0	2.0	1.6								
Glyma.19G212800	Sucrose synthase 2			2.1	1.8						2.9	1.9	1.1
Glyma.20G220800	Germin-like protein subfamily 1 member 10	4.2		2.6									

Table S3 (cont.). Predicted genes commonly found as differentially expressed by transcriptomics (Transcript) and proteomics (Protein) analyses. Statistically significant values of log_2(FC; Fold-change) are given for each gene in its respective treatment (DAI: days after inoculation with *M. incognita*)

Opposite Expression Sense (Transcriptome-Proteome)													
**Genome ID**	**Description**	**30 DAI**				**12 DAI**				**4 DAI**			
		**Transcript**		**Protein**		**Transcript**		**Protein**		**Transcript**		**Protein**	
		**PI 595099**	**BRS 133**	**PI 595099**	**BRS 133**	**PI 595099**	**BRS 133**	**PI 595099**	**BRS 133**	**PI 595099**	**BRS 133**	**PI 595099**	**BRS 133**
Glyma.01G037800	Germin-like protein subfamily 1 member 1	-2.6		4.6	3.5			2.0					
Glyma.01G137700	Pectinesterase inhibitor 17	-3.4	-2.7	1.8				1.4				1.1	
Glyma.02G000700	Sieve element occlusion	-2.4		3.1	2.2			1.6	1.2				
Glyma.02G171600	Peroxidase 53	-2.8		1.6	1.8			1.0					
Glyma.02G272600	Hsp22	-2.3		2.6	1.1			1.4					
Glyma.03G132700	Beta-1,3-glucanase 1-related				3.0					-2.4	-2.4		
Glyma.03G185900	Aquaporin 1	-2.4	-2.2	1.4				1.1					
Glyma.04G136000	Aspartyl protease family protein	-2.1		2.0	1.9			1.1					
Glyma.06G175200	Sieve element occlusion	-2.7		1.7									
Glyma.07G046000	Pectinesterase 2	-2.4		3.1	2.3			1.7					
Glyma.07G047500	Phosphatase DCR2-related	-2.4		1.6	1.1								
Glyma.08G179700	Peroxidase 62				1.4			1.4	1.2		-2.6		
Glyma.08G230000	Hsp20 family	-2.2		1.6	1.8								
Glyma.08G286700	Carbonic anhydrase	-2.9	-3.1	-2.2									1.1
Glyma.09G167000	Sucrose synthase 5	-2.7		1.9									
Glyma.10G155200	Glycoprotein membrane GPI-anchored	-2.5		2.0									
Glyma.10G185500	Sieve element occlusion	-3.3		3.5	2.0			1.7					
Glyma.10G185600	Sieve element occlusion	-4.2	-2.7	1.9	1.4			1.1	1.2				
Glyma.10G185700	Sieve element occlusion	-3.5	-2.6	2.0	1.2			1.3					
Glyma.11G072000	Peroxidase 36	-3.1	-2.3	1.3									
Glyma.11G078400	Peroxidase 5	-2.5		1.9	1.4			1.1					
Glyma.11G080300	Peroxidase 64	-2.7		2.0	2.0			1.4					
Glyma.11G157000	Aspartyl protease family protein	-3.3	-2.3		1.1								
Glyma.13G104800	Malate dehydrogenase	-2.3		1.6									
Glyma.13G178700	Arabinogalactan protein 30	-2.5		1.3	1.9								
Glyma.14G017100	Glycerophosphodiester phosphodiesterase-like protein	-2.1		1.2									
Glyma.15G220800	NADH-cytochrome B5 reductase	-2.6		2.2	2.0			1.2	1.2				
Glyma.16G014100	Pectinesterase 2	-3.2		2.0	2.4								
Glyma.16G060900	Germin-like protein subfamily 1 member 10			2.3	2.1			1.1	1.7	-2.1	-2.4		
Glyma.18G215700	Alpha-galactosidase 1	-2.9		1.1	1.1							-1.0	
Glyma.18G250700	Proprotein convertase subtilisin	-3.6	-2.4	1.1									
Glyma.19G186100	Aquaporin 1	-2.2		1.3									
Glyma.19G234100	Histone H2A	-2.9		1.8									
Glyma.20G052900	Sieve element occlusion	-3.6	-2.5	1.3	1.3								
Glyma.20G145100	Copper amine oxidase	-2.7		1.3									
