# Supplementary material for: Integrated Omic Approaches Reveal Molecular Mechanisms of Tolerance during Soybean and Meloidogyne incognita Interactions
Source: Plants (Basel). 2022 Oct 17;11(20):2744. doi: 10.3390/plants11202744 (PMC9612212; doi:10.3390/plants11202744)
Supplement: Supplementary file 1 [file plants-11-02744-s001.zip › plants-1905218-supplementary/Table S4.pdf]

Table S4. Summary of soybean genes annotated as members of Bet v 1 family

Locus ID	Location	Strand	PFAM ID	# Splice Variants	Gene Length (bp)	Transcript Length (bp) [1]	CDS Length (bp) [1]	Protein Length (a.a.) [1]	Protein MW (kDa) [1]	Protein pI [1]	Tissue (Higher Expression) [2]
Glyma.07G243500	Gm07:42651952..42653004	Reverse	PF00407	3	1,052	916; 895; 859	498; 477; 441	166; 159; 147	17.60; 16.77; 15.53	4.57; 4.69; 4.74	Root
Glyma.07G243600	Gm07:42664435..42665367	Forward	PF00407	1	932	817	477	159	16.76	4.73	Root
Glyma.07G243651	Gm07:42656770..42657698	Forward	PF00407	1	928	771	477	149	16.77	4.69	Root
Glyma.07G243900	Gm07:42675787..42676798	Forward	PF00407	1	1,011	917	483	161	17.98	4.61	Root
Glyma.09G040400	Gm09:3341269..3342584	Forward	PF00407	1	1,315	739	477	159	16.87	4.88	Root
Glyma.09G040500	Gm09:3346523..3347860	Forward	PF00407	1	1,337	909	474	158	17.14	5.34	Root
Glyma.09G040600	Gm09:3362066..3364617	Forward	PF00407	1	2,551	1,006	477	159	16.53	4.68	Root
Glyma.09G040800	Gm09:3373070..3375895	Forward	PF00407	1	2,825	1,191	960	320	34.81	4.91	Flower; Shoot
Glyma.15G145600	Gm15:11983628..11985085	Forward	PF00407	1	1,457	712	477	159	16.86	4.9	N.D. [4]
Glyma.15G145700	Gm15:11987260..11989250	Forward	PF00407	1	1,990	1,587	474	158	17.18	5.59	N.D. [4]
Glyma.15G145800	Gm15:12005923..12006782	Forward	PF00407	1	859	474	474	158	17.15	5.51	N.D. [4]
Glyma.15G145900	Gm15:12011075..12013028	Forward	PF00407	1	1,953	982	465	155	16.14	5.17	Flower; Shoot
Glyma.15G146000	Gm15:12014316..12015314	Forward	PF00407	1	998	844	585	195	21.51	5.07	N.D. [4]
Glyma.15G146100	Gm15:12023556..12024946	Forward	PF00407	3	1,390	807; 737; 651	378	126	13.82	4.71	N.D. [4]
Glyma.15G146400	Gm15:12038706..12041883	Forward	PF00407	1	1,390	807	378	126	13.82	4.71	Flower; Shoot
Glyma.15G146500	Gm15:12042386..12043684	Forward	PF00407	1	1,298	813	483	161	17.71	4.73	N.D. [4]
Glyma.15G146600	Gm15:12047364..12048666	Forward	PF00407	1	1,302	1,179	483	161	17.9	4.79	Root
Glyma.17G030000	Gm17:2202209..2203173	Reverse	PF00407	1	964	870	483	161	17.84	4.65	Root
Glyma.17G030100	Gm17:2209059..2210221	Reverse	PF00407	1	1,162	821	474	158	17.11	4.98	Flower; Leaf; Nodule; Root
Glyma.17G030200	Gm17:2214438..2215414	Reverse	PF00407	1	976	848	477	159	16.76	4.93	Root
Glyma.17G030300	Gm17:2217579..2218493	Forward	PF00407	1	914	786	477	159	16.73	4.92	Root
Glyma.17G030400	Gm17:2220872..2221731	Reverse	PF00407	1	859	773	477	159	16.71	4.8	Root

[1] Data for Each Splicing Variant.
[2] According *Glycine max* Gene Atlas (Phytozome 13).
[3] N.D. – Not Detected.
Each lines highlighted in light green corresponds to member of the same orthogroup.
