# Supplementary material for: Integrated Omic Approaches Reveal Molecular Mechanisms of Tolerance during Soybean and Meloidogyne incognita Interactions
Source: Plants (Basel). 2022 Oct 17;11(20):2744. doi: 10.3390/plants11202744 (PMC9612212; doi:10.3390/plants11202744)
Supplement: Supplementary file 1 [file plants-11-02744-s001.zip › plants-1905218-supplementary/Table S5.pdf]

Table S5. List of primers for RT-qPCR and transgenic tobacco (molecular characterization)

Primers ID	Accession ID	Functional Annotation [1]	Primers Main Features			
			Forward Primer (5'-3')	Reverse Primer (5'-3')	T_M (ºC)	Amplicon Size (bp)
GmNOA1	Glyma.01G018000	Not Annotated 1	ACTTCCACACGAGAGAAGG	AAACTTTGCCAGCATCAGG	60	160
GmFTH1	Glyma.01G124500	Ferritin Heavy Chain (FTH1)	GCCTTGTTTGCGTACTTCG	AGGGAGGACTTGTGATTGG	60	163
GmCYB5	Glyma.03G259600	Cytochrome B5 Isoform A	GTTGACACTTCCACTCTTCC	TAGTACTGCAGGGCAAAGG	60	158
GmUSP	Glyma.04G107900	Universal Stress Protein Family (USP)	AGATTTGAAGCTGGATTCGC	CTTGCTGCTGCTAGTTTCC	60	154
GmALCD	Glyma.04G240800	Alcohol Dehydrogenase Related	GCCATGATCTCAGCATTCG	TCTCCACAACAGAAGGAAGG	60	181
GmNOA2	Glyma.06G056000	Not Annotated 2	TCTGGTGTGTGTTGTTTAGC	AAGCGAAGTGGCAAATACC	60	167
GmBAM5	Glyma.06G301500	ß-Amylase 5 Related	CTCTTATCCACAAAGCCAAGG	CTGGGACATCATTGTACTTGC	60	158
GmBaBl	Glyma.08G128100	Basic Blue Protein	GGACCTTTAACACCGTTACC	AGTCTGATCTGATCCTTCCC	60	178
GmAPHO	Glyma.08G200100	Acid Phosphatase Related	ACACCGTACTCTCTAATATCCC	GAAGCCAAGAGACAACAGC	60	155
GmREMN	Glyma.09G139200	Remorin, N-terminal Region	TTCATCAGTTTCTGCATGGG	GCTCTTCTTTCTTCAGCTTCC	60	156
GmDR4R	Glyma.09G155500	DR4 Protein Related	GTGGTGGATTGAAACTAGGC	GGAGGATTCAGCACAATAAGG	60	188
GmCALN	Glyma.10G147600	Calreticulin and Calnexin	GCTGGTTGAAGAAACATGGG	CGTCTTTGATTCAGCATCGG	60	175
GmAND1	Glyma.13G088700	Annexin D1 Related	GAGAAGGTTGTTCGCTTGG	GGTGGTGTCCTTAACAATAGC	60	162
GmAUXA	Glyma.13G237000	Dormancy/Auxin Associated Protein	CAGAAGACGTTGTCGATGC	TAGACAGTGGGAGTGTTGG	60	188
GmTYR	Glyma.15G071200	Catechol Oxidase/Tyrosinase	TAGGTCAAGGGTTCAGAAGG	AAACCTCTTCCTCCTCTTCC	60	179
GmTRPI	Glyma.15G211500	Trypsin and Protease Inhibitor (Kunitz)	AAACCACGGGATCATACGG	CTGGAACATAACTGGGAGAGG	60	191
GmBetV [2]	Glyma.17G030400	Pathogenesis Related Protein Bet v 1	TCGTTGAGGATGGAGAAACC	GTTTGGTATTCGACAGTGAGC	60	193
GmGST	Glyma.18G190300	Glutathione S Transferase	GAGGAGTTTGGCTTTGTGG	GCATAACTTCGTTGACAACTGG	60	151
GmENOX	Glyma.19G008500	Enone Oxidoreductase	AGAGTGGGAAGTAAAGTGAGG	AAATTGGAGGGTTTGTGAGC	60	152
GmNOA3	Glyma.19G114700	Not Annotated 3	AAGGAGGAGGAGCATAAGG	CTTGACTTTGTCTAGGAACCC	60	168
GmCYP2 [3]	Glyma.12G024700	CYP18-3 Related	CGGGACCAGTGTGCTTCTTCA	CCCCTCCACTACAAAGGCTCG	60	154
GmERF1A [3]	Glyma.05G114900	Translation Factor	GACCTTCTTCGTTTCTCGCA	CGAACCTCTCAATCACACGC	60	161
eGFP [2,4]	MN623123.1 [5]	Enhanced Green Fluorescent Protein	ACTTCAAGATCCGCCACAAC	GAACTCCAGCAGGACCATGT	60	179
NtACT1 [2,3]	U60489.1 [5]	*Nicotiana tabacum* Actin	AGCAAGGAAATTACCGCATTAGC	ACCTGCTGGAATGTGCTGAGA	60	125
NtL25 [2,3]	L18908.1 [5]	*Nicotiana tabacum* Ribosomal Protein L25	GCTAAGGTTGCCAAGGCTGTCAAG	GCACTAATACGAGGGTACTTGGGGTTTC	60	134

[1] For soybean genes, the assembly version used was *Glycine max* Wm82.a2.v1 (Phytozome 13).
[2] Transgenic Tobacco Molecular Characterization
[3] Housekeeping/Reference Genes.
[4] Tobacco Transformation Marker.
[5] Accession ID from NCBI database.
